# Quantitative Trait Loci for Freezing Tolerance in a Lowland x Upland Switchgrass Population

**DOI:** 10.3389/fpls.2019.00372

**Published:** 2019-03-29

**Authors:** Hari P. Poudel, Millicent D. Sanciangco, Shawn M. Kaeppler, C. Robin Buell, Michael D. Casler

**Affiliations:** ^1^Department of Agronomy, University of Wisconsin–Madison, Madison, WI, United States; ^2^Department of Plant Biology, Plant Resilience Institute, and MSU AgBioResearch, Michigan State University, East Lansing, MI, United States; ^3^U.S. Dairy Forage Research Center, United States Department of Agriculture-Agricultural Research Service, Madison, WI, United States

**Keywords:** quantitative trait loci, freezing tolerance, LT-50, *Panicum virgatum* (switchgrass), single nucleotide polymorphism

## Abstract

Low-temperature related abiotic stress is an important factor affecting winter survival in lowland switchgrass when grown in northern latitudes in the United States. A better understanding of the genetic architecture of freezing tolerance in switchgrass will aid the development of lowland switchgrass cultivars with improved winter survival. The objectives of this study were to conduct a freezing tolerance assessment, generate a genetic map using single nucleotide polymorphism (SNP) markers, and identify QTL (quantitative trait loci) associated with freezing tolerance in a lowland × upland switchgrass population. A pseudo-F_2_ mapping population was generated from an initial cross between the lowland population Ellsworth and the upland cultivar Summer. The segregating progenies were screened for freezing tolerance in a controlled-environment facility. Two clonal replicates of each genotype were tested at six different treatment temperatures ranging from −15 to −5°C at an interval of 2°C for two time periods. Tiller emergence (days) and tiller number were recorded following the recovery of each genotype with the hypothesis that upland genotype is the source for higher tiller number and early tiller emergence. Survivorship of the pseudo-F_2_ population ranged from 89% at −5°C to 5% at −15°C with an average LT_50_ of −9.7°C. Genotype had a significant effect on all traits except tiller number at −15°C. A linkage map was constructed from bi-allelic single nucleotide polymorphism markers generated using exome capture sequencing. The final map consisted of 1618 markers and 2626 cM, with an average inter-marker distance of 1.8 cM. Six significant QTL were identified, one each on chromosomes 1K, 5K, 5N, 6K, 6N, and 9K, for the following traits: tiller number, tiller emergence days and LT_50_. A comparative genomics study revealed important freezing tolerance genes/proteins, such as COR47, DREB2B, zinc finger-CCCH, WRKY, GIGANTEA, HSP70, and NRT2, among others that reside within the 1.5 LOD confidence interval of the identified QTL.

## Introduction

Low-temperature related abiotic stress is an important factor affecting winter survival in lowland switchgrass (*Panicum virgatum* L.) when grown in northern latitudes in the United States (Lemus et al., 2002; Milano et al., 2016). Lowland switchgrass has generated considerable interest due to its higher biomass yield associated with late flowering compared to the upland ecotype (Wullschleger et al., 2010; Casler, 2012; Casler and Vogel, 2014). However, when planted outside the range of natural adaptation, lowland populations exhibit low winter survival, resulting in substantive reduction in biomass production and stand loss. Lowland cultivars planted in northern Wisconsin had an average biomass yield of 4.2 Mg ha^−1^ yr^−1^ compared to 15 Mg ha^−1^ yr^−1^ in Oklahoma with winter survival in Wisconsin nearly 50% less than in Oklahoma (Casler et al., 2004). On the other hand, lowland cultivars may have doubled the biomass yield of upland cultivars when planted in southern locations (Casler, 2012).

For the sustainable and economical use of switchgrass as a biomass crop, a minimum of 20 Mg ha^−1^ yr^−1^ dry matter biomass yield that survives multiple years is desirable (Taliaferro, 2002; Casler, 2012). To achieve this goal, two traits mainly form the focus in switchgrass breeding programs: late flowering to extend the growing season and winter survivorship to ensure stand longevity. Three strategies are being employed to accomplish this goal: (1) the use of upland × lowland hybrids (Vogel et al., 2014), (2) selection for late flowering within northern-adapted upland germplasm, and (3) selection for winter survivorship within late-flowering but unadapted southern lowland germplasm. Identification of freezing tolerance QTL using the first strategy is the focus of this research.

Switchgrass prepares for senescence and dormancy at the onset of winter and as the temperature and photoperiod begin to decrease (Sarath et al., 2014). During dormancy, the plant suffers from low-temperature stress, which may be manifested as water-logging, ice-encasement, anoxia, or desiccation. However, freezing stress is generally accepted as the single component explaining the variation in winter survival (Pulli et al., 1996). Freezing damage is caused by osmotic dehydration triggered by extracellular ice formation, which leads to cell lysis and, eventually, death of the plant (Steponkus, 1984; Guy, 1990). Cold acclimation or hardening at low but non-freezing temperatures has often resulted in increased freezing tolerance. The ability of plants to survive freezing temperatures is largely dependent upon their ability to cold acclimate, which triggers an increase in production of cryoprotectant molecules, such as sugars, proline, and, serine, as well as changes in the lipid composition of membranes (Téoulé and Géry, 2014). C-repeat binding factors (CBF) are thought to have key roles in regulating cold-responsive genes (COR). The CBF transcription factors recognize the dehydration-responsive element (DRE) in the regulatory region of COR genes for conferring freezing tolerance (Stockinger et al., 1997). Freezing tolerance has been extensively studied as a complex quantitative trait, but some freezing tolerance genes in the model plant *Arabidopsis thaliana* have been identified as having major effects on phenotype (Warren et al., 1996; Thomashow, 1999; Alonso-Blanco et al., 2005). In crop species, genes involved in freezing tolerance have been reported by Vágújfalvi et al. (2003); Francia et al. (2007), Alm et al. (2011), and Shirasawa et al. (2012). However, there are no known studies in switchgrass that have identified quantitative trait loci (QTL) for freezing tolerance.

In this study, a pseudo-F_2_ population, derived from a cross between a lowland and upland ecotype, was used. Identification of QTL underlying freezing tolerance and their use with marker-assisted selection will help in the development of freezing-tolerant lowland switchgrass cultivars with improved biomass yield. The objectives of this study were to: (1) conduct a freezing-tolerance screening of a pseudo-F_2_ switchgrass population in controlled environment chambers, (2) construct a genetic map using single nucleotide polymorphism (SNP) markers generated through exome-capture sequencing, and (3) identify QTL associated with freezing tolerance in a lowland x upland switchgrass mapping population. Our hypothesis was that upland cultivars contain alleles favorable for freezing tolerance, as indicated by a higher number of tillers and early emergence following exposure to freezing stress.

## Materials and Methods

### Mapping Population

The initial F_1_ hybrid was a cross between Ellsworth, a late flowering lowland switchgrass (*Panicum virgatum* L.) population with low winter survival (origin: Ellsworth, KS, USDA hardiness zone 6a) and Summer, an early flowering upland ecotype with higher winter survival (origin: Nebraska City, NE, USDA hardiness zone 5b) made in the glasshouse in 2012. The synchronization of flowering was supplemented by the use of fluorescent lights to adjust the photoperiod-mediated process, a method similar to that by Castro et al. (2011). Two random F_1_ individuals were crossed to generate the ELLSU-17 pseudo-F_2_ testcross population of 341 progenies. A detailed explanation about the development of mapping population can be found in Tornqvist et al. (2018). These individuals were transplanted near DeKalb, IL in July 2014 for a separate flowering time study (Tornqvist et al., 2018). After the flowering time study had commenced, 208 F_2_ testcross progenies that survived 2 years of field conditions were used to generate phenotypic data in this study. The remaining progenies were either dead due to transplanting stress or winter stress in field.

During the first week of October 2016, rhizomes from 208 ELLSU-17 individuals, along with their parents and grandparents at DeKalb, IL, United States were dug, wrapped in plastic and transported to Madison, WI. The rhizomes were in the early dormant stage. Dormant rhizomes were used to simulate normal conditions for switchgrass plants, undergoing the cold hardening process at this latitude. These rhizomes were randomly arranged in the upright position into three wooden cold frames (3.6 m long and 1.8 m width), located immediately outside the greenhouse where they were intended to be evaluated. The rhizomes were left in cold frames for about 2 months to facilitate hardening and cold acclimation. There was no snow fall during this time and there were periods of below-zero minimum air temperature during the acclimation period (Madison Dane County Regional Airport, WI, United States)^1^ (Supplementary Figure S1). For those nights when the minimum temperature was expected to be below zero, the cold frames were covered by plastic tarps, maintaining minimum temperatures between 0 and 10^°^C during the acclimation period. After hardening, rhizomes from each individual were divided into 28 clonal ramets, such that each ramet consisted of two tiller buds. The clones were transplanted to 5-cm containers containing a commercial potting mixture (Pro Mix^®^ HP Mycorrhizae^TM^, Premier Tech Horticulture ltd., Rivière-du-Loup, QC, Canada), watered to saturation and then stored in a cooler at 4°C until the temperature trials were initiated.

### Experiment Design for Freezing Stress

Vegetative ramets from each grandparent, parent, and progeny genotype were randomly assigned to one of the six treatment temperatures: −15, −13, −11, −9, −7, −5°C in a randomized complete block design with four blocks. The freezing screening was conducted at the University of Wisconsin-Madison Biotron controlled environment facility^2^. The four blocks were tested in two cycles at an interval of 1 week (beginning on 1st or 8th of December 2016), due to space limitations, with two blocks per cycle. To minimize the stress due to larger temperature differences, a staged cooling rate protocol as described by Peixoto and Sage (2016) was used.

All sample transplants were initially kept at 4°C in a freezing-capable room. The temperature was lowered in stages to the treatment temperature at a cooling rate of −1°C hr^−1^. At each treatment temperature, the samples were incubated for 24 h. Each 24-h incubation period represents a thermal stage. Following the conclusion of each thermal stage, plants were transferred to a ‘thawing room’, which was pre-cooled at the treatment temperature. The samples were thawed to 4°C at the heating rate of +1°C hr^−1^ and then transferred to a ‘holding room’ which was initially set at 4°C. When all the transfers were completed, the temperature of the ‘holding room’ was increased by +1°C day^−1^ to 14°C. Finally, all treated samples were transferred to a greenhouse and allowed to regrow. The greenhouse temperature was gradually increased by 1°C day^−1^ and maintained at 24°C with a 12-h photoperiod using GE lucalox^®^ 27187 high pressure sodium lights for 6 weeks. Watering was done once or twice per day depending on the moisture conditions. Tissue viability was assessed on each sample with the rhizome regrowth method by Peixoto and Sage (2016). Tiller emergence (days) was recorded starting with 7 days of recovery as the number of days prior to initial emergence from the soil. The tiller emergence days were reported every alternate day. Tiller number per plant was recorded after 30 days of recovery.

### Statistical Analysis

Best linear unbiased predictors (BLUP) for QTL mapping were calculated for tiller number at each treatment temperature using a random-effects linear model (Equation 1) in R package lme4 (Bates et al., 2014).

(1) $$y_{\mathrm{ijk}}=\mu +c_{i}+g_{i}+b_{k(i)}+{\left(c\;x\;g\right)}_{\mathrm{ij}}+e$$

Where y_ijk_ is the predicted response, μ is grand mean, c is the effect of cycle, g is the effect of genotype, b is the effect of block nested within the cycle, and e is residual error. The cycle is included in the model to account for the differences in the acclimation period between the two treatment cycles, such that the samples in cycle two received seven extra days of cold acclimation at °4C.

A combined single model was used to calculate (BLUPs) for tiller emergence days based on the random-effects linear model (Equation 2) in R package lme4 (Bates et al., 2014). Transplants which did not emerge were removed from the analysis because of the difficulty in quantifying the days of emergence.

(2) $$y_{\mathrm{ijkl}}=\mu +t_{i}+c_{j}+g_{k}+b_{l(j)}+{\left(t\;x\;c\right)}_{\mathrm{ij}}+{\left(c\;x\;g\right)}_{\mathrm{jk}}+{\left(t\;x\;g\right)}_{\mathrm{ik}}+{\left(c\;x\;t\;x\;g\right)}_{\mathrm{ijk}}+e$$

where, terms are as defined in Equation (1) and t is the effect of temperature.

Survivorship data with binary response values were used for calculation of LT_50_ using probit analysis in SAS 9.4 (SAS, 2015) separately for each cycle. Genotypes with zero phenotypic variance across all treatment temperatures within each cycle were not estimable, and thus were excluded from the analysis. The probit procedure generated a table of predicted percentage survival at each temperature and the temperature corresponding to 50% survival was used as the estimates of LT_50_ of each genotype. Further, genotypes with LT_50_ deviating by ±10°C away from the range of treatment temperature were removed as outliers. Finally, the remaining 202 genotypes (including parental) were used for mixed model analysis and calculation of BLUP using the following model:

(3) $$y_{\mathrm{ijk}}=\mu +c_{i}+g_{j}+{\left(c\;x\;g\right)}_{\mathrm{ij}}+e$$

where terms are as defined in Equation (1).

Broad-sense heritability was calculated from estimated variance components of genotype (Vg) and prediction error variance (PEV) as H = 1- (PEV/Vg) (Clark et al., 2012), which is equivalent to genotype-mean heritability.

### Genotyping

Exome capture sequence data was generated using the NimbleGen SeqCap EZ Switchgrass Exome probe, as described previously (Tornqvist et al., 2018). Raw reads were trimmed using Cutadapt v1.9.1 (Martin, 2011), and their quality was assessed before and after trimming using FastQC v0.11.5^3^ and MultiQC v1.0 (Ewels et al., 2016). Samples with a very low number of reads were excluded in subsequent analyses. The paired reads for each sample were then aligned to the switchgrass reference genome (Pvirgatum_450_v4.0.hardmasked.fa) using BWA v0.7.15 (Li et al., 2009) and piped to SAMTools v1.3.1 (Li et al., 2009). Reads were further sorted and indexed using SAMTools v1.3.1 (Li et al., 2009). Duplicate reads were marked and removed using Picard v2.7.2^4^. Local realignment was performed using the Realigner Target Creator and Indel Realigner tools from GATK v3.7.0 (McKenna et al., 2010) to minimize the number of mismatching bases across all reads. Pileup files were then generated using SAMTools v1.3.1 (Li et al., 2009) mpileup command with BAQ disabled and map quality adjustment disabled. Read data was extracted for 5,596,351 bi-allelic loci previously identified from three diversity panels (Acharya, 2014; Evans et al., 2014; Evans et al., 2018), initially mapped to v3 (Pvirgatum_383_v3.1) and lifted over to v4 of the reference genome, and filtered to remove any alleles not present in the original dataset.

The genotype dosages were called at each locus using EM algorithm of Martin et al. (2010) implemented in R. The data was further filtered for polymorphic markers and minor-allele frequency greater than 1/2N, where N is the number of individuals. Markers with more than 30% missing data were excluded from the analysis. Eight independent samples of each grandparent and parent were submitted for genotyping and only markers that were concordant at six or more of these samples were selected. We used χ^2^ goodness-of-fit tests for the 1:2:1 distributed marker to identify putative F_2_-type markers and 1:1 distributed markers to identify backcross-type (BC-type) markers with a threshold of *p* > 0.01. Further filtering was done by constructing 5-Kb bins and randomly selecting one marker from each bin, due to software limitations. With chromosome 1K and 3N, markers segregating with a χ^2^ *p* > 0.001 were used to fill out large gaps in the map, supplementing the sequence information that is publicly available.

### Linkage Map and QTL Analysis

The linkage map was constructed using Join Map software^®^ version 4.1 (Van Ooijen, 2006). The markers were entered as a cross-pollinated (CP) population with three categories of codominant SNP markers: markers heterozygous for both F1 parents (hk × hk, F2 type), markers heterozygous in one parent and homozygous in the other parent (lm × ll, BC type 1) and the reverse of BC type 1 (nn × np, BC type 2). The independent LOD parameter threshold, ranging from 2 to 16 at a step of one, was used to group the markers into linkage groups (LGs). We used switchgrass reference genome V4.1 information to remove any markers from the linkage groupings which did not fit the chromosome grouping. The markers were ordered using the regression mapping method by selecting the Kosambi mapping function.

The QTL analysis was conducted for all traits in R using the stepwise-QTL model fitting method, as implemented in the Rqtl package (Broman et al., 2003). All QTL scans were performed using the normal model and Haley-Knot regression method on a dense 2-cm grid using the cal.genoprob function. The LOD threshold value and LOD penalties for each trait were calculated based on 1000 permutations of batch size 20 using the scantwo function. An optimized QTL model was determined using stepwise scan and the final model was fitted using fitqtl function similar to the method of Milano et al. (2016) and Tornqvist et al. (2018). The final marker data, genetic map and phenotypic BLUP data are available as Supplementary Tables S1–S3 respectively.

## Results

### Phenotypic Analysis and Heritability of Traits

There was a significant effect of genotype (*p* < 0.01) for all traits except for tiller number measured at −15°C (Table 1). Genotype x cycle interaction was only significant for tiller emergence days and tiller number at −11°C. The genotype × temperature interaction was significant (*p* < 0.01) for tiller number indicating that QTL analysis should be conducted separately for each temperature. However, transplants which did not emerge were removed, thus QTL analysis for tiller emergence at each temperature were not performed even though genotype × temperature interaction was significant (*p* < 0.01). The effect of cycle was significant for tiller emergence and tiller number combined analysis, probably because of the extra 7 days of cold acclimation in the samples used in cycle 2.

**Table 1:** Mixed model analysis of variance for fixed effects associated with freezing tolerance for phenotypic traits of switchgrass (tiller emergence, LT_50_ and tiller number).

Traits	Sources of variation	df	*F*-value	Significance
Tiller emergence	Cycle (C)	1	163.25	^∗∗∗^
(days)	Temperature (T)	5	12.82	^∗∗∗^
	Genotype (G)	207	3.47	^∗∗∗^
	G × C	202	1.50	^∗∗∗^
	G × T	758	1.31	^∗∗∗^
	G × C × T	568	1.10	
Tiller number	Cycle (C)	1	5.39	^∗^
	Temperature (T)	5	617.82	^∗∗∗^
	Genotype (G)	207	6.00	^∗∗∗^
	G × C	202	1.69	^∗∗∗^
	G × T	1035	1.59	^∗∗∗^
	G × C × T	1007	1.08	
LT-50	Cycle (C)	1	0.05	
	Genotype (G)	201	2.08	^∗∗∗^
	G × C	193		
Tiller number	Cycle (C)	1	7.12	^∗∗^
(−5°C)	Genotype (G)	207	1.87	^∗∗∗^
	G × C	202	1.19	
Tiller number	Cycle (C)	1	1.43	
(−7°C)	Genotype (G)	207	1.85	^∗∗∗^
	G × C	202	1.14	
Tiller number	Cycle (C)	1	1.91	
(−9°C)	Genotype (G)	207	2.51	^∗∗∗^
	G × C	202	1.12	
Tiller number	Cycle (C)	1	15.87	^∗∗∗^
(−11°C)	Genotype (G)	207	3.76	^∗∗∗^
	G × C	201	1.52	^∗∗∗^
Tiller number	Cycle (C)	1	1.18	
(−13°C)	Genotype (G)	207	2.72	^∗∗∗^
	G × C	201	1.11	
Tiller number	Cycle (C)	1	4.75	^∗^
(−15°C)	Genotype (G)	207	1.01	
	G × C	201	1.04	

*^∗^Significant at the 0.05 probability level. ^∗∗^Significant at the 0.01 probability level. ^∗∗∗^Significant at the 0.001 probability level.*

**FIGURE 1:**
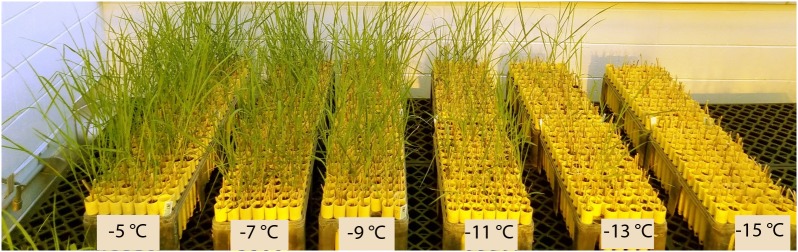
Greenhouse planting arrangement illustrating the effect of temperature on regrowth of switchgrass after freezing stress. Beginning from the left, the racks represent survivorship and recovery from treatment temperatures –5, –7, –9, –11, –13, and –15°C. All racks belong to the same replicate and each column of racks contains a maximum of 192 random genotypes.

The average survival of rhizomes at the control temperature (4°C) was 92%, which was similar to the sprouting rate (>90%) of switchgrass rhizomes at the untreated temperature mentioned by Peixoto and Sage (2016). Overall survival after freezing decreased from 89% at the highest treatment temperature (−5°C) to 5% at the lowest treatment temperature (−15°C) (Table 2). Figure 1 illustrates the differences in vigor and survivorship across the six temperature treatments. Because of low survivorship (<5%) accompanied with a non-significant genotype effect (Table 1), tiller number at −15°C was excluded from subsequent QTL analysis. Tiller emergence and LT_50_ had broad-sense heritability (H) values of 0.54 and 0.51, respectively (Table 2). Heritability estimates for tiller number increased from 0.33 to 0.59 with a decrease in treatment temperature from −5 to −13°C and dropped to 0 at −15°C due to the extreme mortality.

**Table 2:** Best linear unbiased predictors (BLUP) mean survival, and heritability for phenotypic traits (tiller emergence, LT_50_ and tiller number) of switchgrass parents, grandparents, and the F_2_ population.

	Tiller emergence (days)	LT_50_ (°C)	−5°C	−7°C	−9°C	−11°C	−13°C	−15°C
Ellsworth aaa	4.9	−8.1	1.2	0.9	0.5	0.7	0.2	<0.1
Summer bbb	4.1	−10.3	1.3	1.3	1.2	1.7	0.3	<0.1
ELLSU-1 (F_1_)	4.5	−9.3	1.4	1.3	1.2	0.8	0.3	<0.1
ELLSU-7 (F_1_)	7.0	−10.8	1.3	1.4	1.2	0.8	0.8	<0.1
ELLSU-17 (F_2_) ^†^	5.6	−9.7	1.4	1.2	1.1	0.9	0.4	<0.1
	(3.5–9.1)	(−13.3––4.8)	(0.9–1.9)	(0.8–1.7)	(0.5–2.3)	(0.4–2.3)	(0.2–1.4)
Survival (%)			89	84	76	70	33	5
Heritability	0.54	0.51	0.33	0.38	0.55	0.59	0.59	0.00

*^†^Mean of BLUP (range in parenthesis).*

**FIGURE 2:**
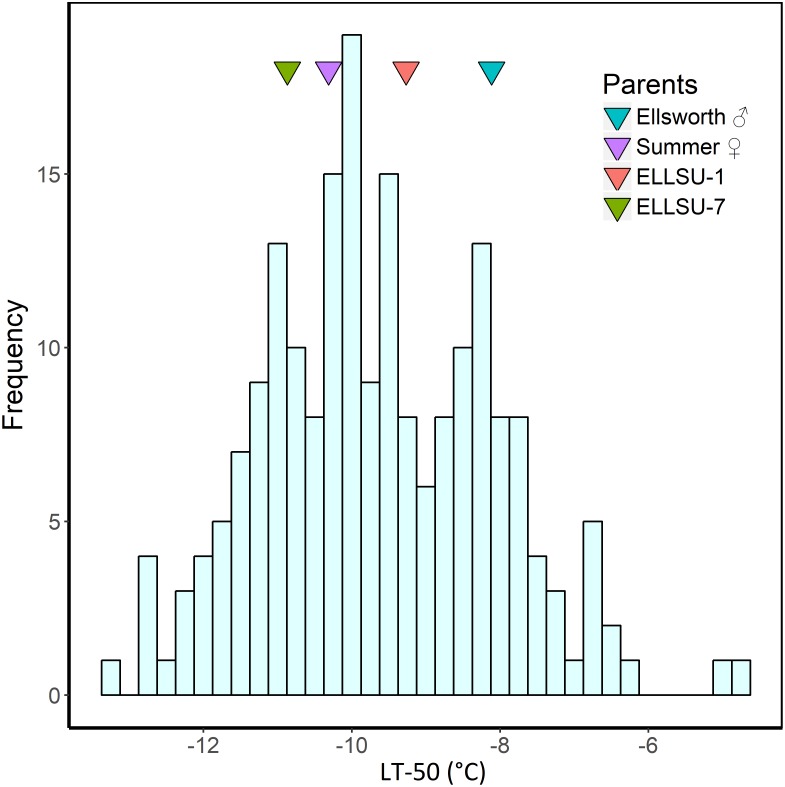
Histogram of LT_50_ best linear unbiased predictors (BLUP) in F_2_ progenies derived from the ELLSU-17 lowland × upland switchgrass cross. The LT_50_ BLUP of the grandparents and parents are shown by colored triangles within the plot.

The BLUP estimates indicated that Ellsworth (lowland grandparent) required more time to recover from freezing than Summer (upland grandparent) and F_2_ progenies, on average, were later emerging than both grandparents (Table 2). Similarly, the LT_50_ for Ellsworth was 2.2°C greater than for Summer, clearly indicating the superior freezing tolerance of the upland ecotype. The distribution of LT_50_ BLUP in the F_2_ population was normal and continuous with mean 9.7°C and variance 4.6 (Figure 2). The average value for the F_2_ individuals was close to that for the upland parent, which had a mean value of −10.3°C. Summer also had higher tiller numbers compared to Ellsworth following recovery from each freezing temperature, an effect that was fairly constant across temperatures, except for the −15°C temperature.

Tiller numbers at each temperature above −15°C were all positively correlated on a genotypic basis within the F_2_ population (Table 3). As such, values of LT_50_ were negatively correlated with tiller number BLUP for each of these five temperatures. Tiller emergence time was not correlated with any of these other traits, except for a small negative correlation with tiller number at −5°C.

**Table 3:** Genotypic correlation coefficients (*r*) for phenotypic traits estimated using best linear unbiased predictors (BLUP) in the F_2_ population derived from the ELLSU-17 lowland × upland switchgrass cross.

	LT_50_	Tiller emergence (days)	Tiller number (−5°C)	Tiller number (−7°C)	Tiller number (−9°C)	Tiller number (−11°C)
Tiller emergence (days)	−0.03					
Tiller number (−5°C)	−0.51^∗∗∗^	−0.17^∗∗^				
Tiller number (−7°C)	−0.51^∗∗∗^	−0.05	0.56^∗∗∗^			
Tiller number (−9°C)	−0.41^∗∗∗^	−0.07	0.43^∗∗∗^	0.41^∗∗∗^		
Tiller number (−11°C)	−0.48^∗∗∗^	−0.03	0.32^∗∗∗^	0.41^∗∗∗^	0.29^∗∗∗^	
Tiller number (−13°C)	−0.64^∗∗∗^	−0.02	0.31^∗∗∗^	0.37^∗∗∗^	0.31^∗∗∗^	0.45^∗∗∗^

*^∗∗^Significant at the 0.01 probability level. ^∗∗∗^Significant at the 0.001 probability level.*

### Linkage Mapping and QTL Detection

**FIGURE 3:**
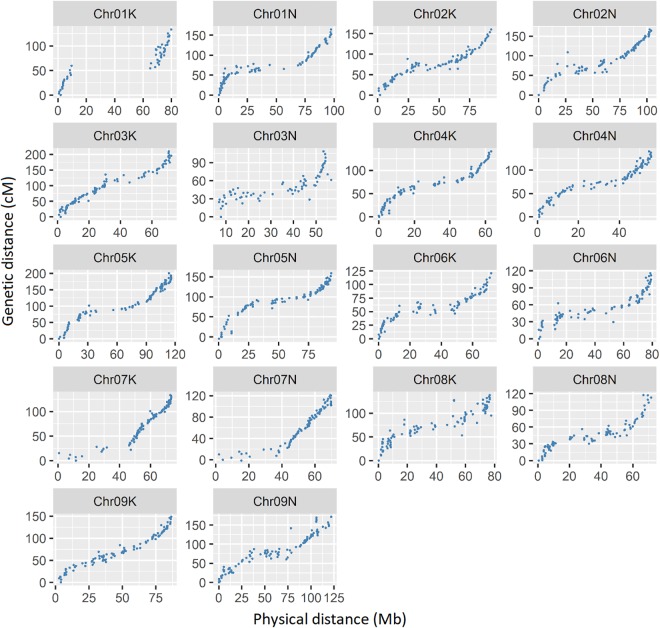
Relationship between physical map distance based on *Panicum virgatum* v4.1 in MB with genetic distance in cM. Each dot represents a single SNP marker. The simple correlation (r) between the physical and genetic maps, averaged over all chromosomes, is 0.93.

A total of 1618 SNP markers were grouped into 18 linkage maps, corresponding to 18 chromosomes of tetraploid switchgrass (Supplemental Table 2). The total map length was 2626 cM with an average inter-marker distance of 1.8 cM, which is within the comparable range of previous studies (Okada et al., 2010; Liu et al., 2012; Serba et al., 2013; Lowry et al., 2015; Milano et al., 2016; Tornqvist et al., 2018). The Pearson correlation coefficient between physical position based on V4.1 of the switchgrass reference genome and the genetic map position, averaged across all chromosomes, was 0.93 (Figure 3). The lowest correlation was on chromosome 3N (*r* = 0.82) and the highest correlation was in chromosome 9K (*r* = 0.98). The shortest map size was in chromosome 3N (109 cM) and longest in chromosome 3K (210 cM). The number of SNP markers on each linkage group ranged from 48 on chromosome 1K to 99 on chromosome 2K and 9K.

**FIGURE 4:**
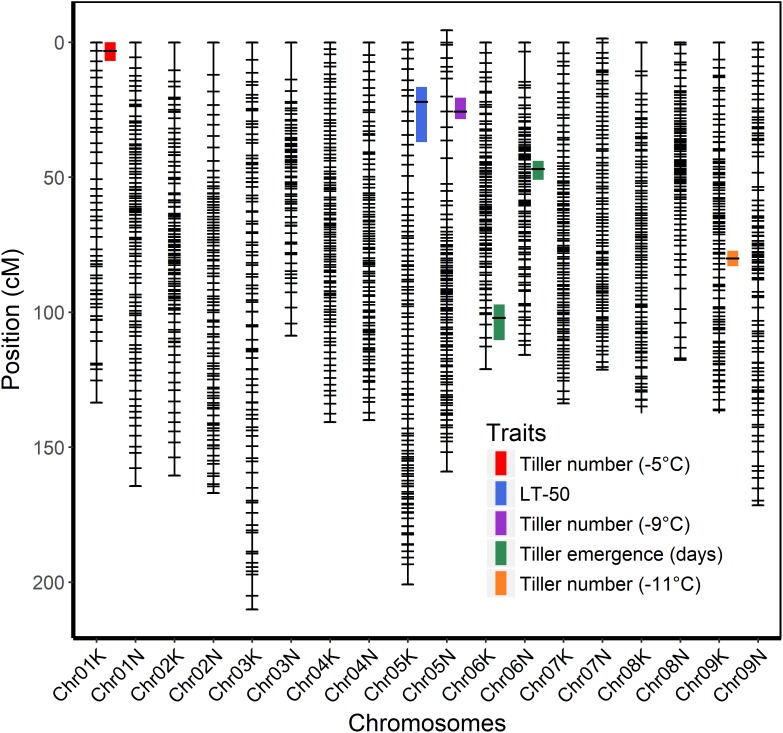
Genetic linkage map of the ELLSU-17 lowland x upland switchgrass population based on reference genome V4.1 and showing QTL with 1.5 LOD confidence interval plotted to the right of respective chromosomes.

The genetic map was constructed using genotypic data of all available 341 F_2_ individuals while only 208 F_2_ individuals that survived the field conditions were used to measure phenotypes for detecting associations with markers. A total of six QTL were identified using stepwise model selection with a genome-wide threshold level of 0.05 (Table 4 and Figure 4). Two QTL for tiller emergence and one each for LT_50_, tiller number at −5°C, tiller number at −9°C and tiller number at −11°C were identified on chromosomes 6K, 6N, 5K, 1K, 5N, and 9K, respectively. The percentage of phenotypic variation explained by QTL (PVE) ranged from 9 to 16 % (Table 4) and the largest PVE was observed for tiller emergence on chromosome 6N at position 46.9 cM. No significant QTL were found for tiller number at −7 or −13°C and none of the detected QTL were observed for more than one trait. This latter observation likely reflects the strong genotype x temperature interaction detected in the mixed model ANOVA.

**Table 4:** Quantitative trait loci (QTL) identified as significant by LOD in the F_2_ population derived from the ELLSU-17 lowland × upland switchgrass cross.

QTL^†^	Trait	Physical position (bp)^¶^	LOD	1.5 LOD interval (cM)	PVE^‡^	Transcript name^§^	PANTHER gene description^§^
1K.3	Tiller number (−5°C)	731672	5.0	1–6	10.4	Pavir.1KG002600	Glycerate dehydrogenase/ Hydroxypyruvate reductase/D-glycerate
5K.22	LT_50_	6808951	6.1	17–36	12.9	Pavir.5KG046800	Phosphoribosylaminoimidazole carboxylase/AIR carboxylase
5N.26	Tiller number (−9°C)	4484505	5.7	21–27	11.9	NA	NA
6K.102	Tiller emergence (days)	68440249	4.9	98–109	8.7	Pavir.6KG368900	*S*-Adenosyl-L-Methionine-Dependent Methyltransferase-Like Protein
6N.47	Tiller emergence (days)	35858523	8.4	46–48	15.8	Pavir.6NG191500	Flavodoxin related
9K.80	Tiller number (−11°C)	58669384	5.6	78–82	11.7	Pavir.9KG370400	NA

*NA, Information not available. ^†^QTL name by chromosome and peak position (cM). ^¶^ Physical base position corresponding to Switchgrass reference genome V4.1. ^‡^Percentage phenotypic variance explained. ^§^ Transcripts name and gene description (http://www.phytozome.net).*

### Allelic Effects

**FIGURE 5:**
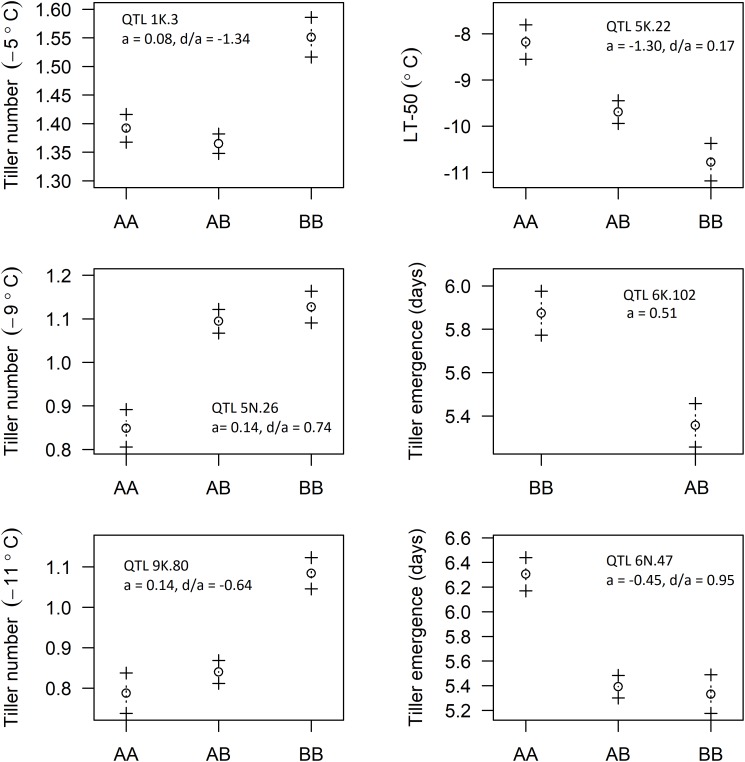
Effect of allelic substitution on identified QTL: AA represents “Ellsworth” grandparent, BB represents “Summer” grandparent, and AB represents hybrid type with one allele coming from each of the grandparents (a, additive effect; d, dominance effect).

Five of the six identified QTL were of the F_2_ type and the other was the backcross (BC) type. The genotypes of identified QTL were coded such that the “A” allele code was assigned to Ellsworth and the “B” allele code to Summer. The additive effects (a) for the putative QTL observed for LT_50_, tiller emergence, and tiller number at −5, −9, and −11°C are shown in Figure 5. Additive effects were positive for tiller number at all three temperatures and negative for LT_50_, indicative of greater freezing tolerance from the Summer grandparent. Additive effects for the two putative QTL associated with tiller emergence were opposite in sign, indicating neither Summer nor Ellsworth was an exclusive source of alleles for more rapid emergence following freezing. Dominance effects were all near to or less than 1, indicating complete or incomplete dominance gene action for all six of these putative QTL.

## Discussion

### Freezing Tolerance Screening

The upland ecotype of switchgrass is highly tolerant of freezing temperatures, as evidenced by several previous studies. The upland cultivar Pathfinder was progressively more freezing tolerant as the hardening process was allowed to progress from September through December, with an LT_50_ ranging from −4°C to −22°C during this time period (Hope and McElroy, 1990). Rhizomes of upland switchgrass have been reported to survive in the field after exposure to air temperatures as low as −20°C (Ichizen et al., 2002; Sage et al., 2015). The observed LT_50_ of Summer switchgrass (upland) in our study was well within these ranges, averaging −10.3°C (Figure 2). This coincides well with field-based survivorship data for Summer, which showed ground cover > 82% for several locations within USDA hardiness zones 3b to 5b (Vogel et al., 2014). LT_50_ is regarded as one of the most reliable measures of freezing tolerance for screening plant genotypes (Båga et al., 2007; Skinner and Garland-Campbell, 2014), and our results suggest that LT_50_ behaved as expected, based on the origin of the two parents, Summer and Ellsworth. As such, we expected most favorable alleles in the progeny to have originated from the Summer grandparent. The fact that some favorable alleles originated from the Ellsworth population was indicative of two phenomena: (1) lowland population of switchgrass contain favorable alleles for freezing tolerance, but likely at low frequencies and (2) upland genotypes of switchgrass likely have favorable alleles for most loci involved in freezing tolerance, but not all loci. Support for the first of these phenomena comes from simple breeding studies, in which the adaptation of lowland populations has been broadened by selection for winter survivorship (Casler and Vogel, 2014; Casler et al., 2018). For the second phenomenon, Summer originates from the southern boundary of the natural adaptation zone of the upland ecotype, which may explain why its genotypes do not require favorable alleles at all QTL for freezing tolerance.

We observed almost complete mortality in the pseudo-F_2_ population at −15°C and 50% mortality at −9.7°C. These results suggest that breeding freezing-tolerant lowland switchgrass for USDA plant hardiness zones 3–5, corresponding to mean minimum temperature of −40 to −23°C (USDA, 2012) will be challenging. While snow cover can be an effective insulator for lowland switchgrass, allowing moderate survivorship at some northern locations (Casler et al., 2018), snow cover is unreliable and there is a clear need for germplasm that has improved freezing tolerance. The high mortality in our experiment could be due to cumulative freezing stress on the samples, as a result of the staged freezing protocol (Peixoto and Sage, 2016). Development of late-flowering and freezing-tolerant cultivars can be best accomplished by exploiting freezing-tolerant alleles from upland cultivars and increasing their frequency in upland x lowland crosses by phenotypic recurrent selection, as was done to develop the cultivar Liberty. In this cultivar, field-based selection was effective, as evidenced by >93% ground cover compared to 10–82% for its lowland parent Kanlow (Vogel et al., 2014). However, because the phenotypic recurrent selection for the development of Liberty required almost 20 years, more rapid and reliable approaches are required to develop better cultivars in a shorter period of time. Therefore, QTL identified in this study could serve as useful genetic resources for marker-assisted breeding to accelerate the breeding cycle and ultimately increase the selection efficiency *per se* (Collard and Mackill, 2008).

### Construction of the Genetic Linkage Map

The pseudo-F_2_ population in this study was developed from a cross of two sister F_1_ plants and is highly heterozygous. One way to map this type of population with known phasing would be to use only markers that are F2-type in the parents and BC-type in the grandparents (Braun et al., 2017). However, the use of only F2-type markers for mapping in our population led to unexpected groupings of markers, large gaps between markers and clustering of markers to the tails of the chromosome. Therefore, both F2 and BC-type markers were used by treating this as a CP-type population in Joinmap 4.1 (Van Ooijen, 2006), similar to Milano et al. (2016) and Tornqvist et al. (2018). This strategy may be less powerful than the classical QTL mapping as conducted in inbred populations because the markers and alleles may be in the different states and linkage phases (Boopathi, 2012). Nevertheless, this method has still proven useful to group markers to the appropriate linkage groups, detect significant QTL, and to compute the magnitude and direction of QTL effects in the cross-pollinated population.

In this study, the selected markers were perfectly grouped into 18 linkage groups, corresponding to 18 chromosomes of tetraploid switchgrass (Evans et al., 2015) and spanning a total map length of 2626 cM. While the map size in this study is relatively longer than the previously published genetic map in switchgrass, there is a high level of concordance with the physical map (Figure 3). The map length of the same population used in this study (ELLSU-17) by Tornqvist et al. (2018), based on an earlier version of the genome (V1.1 of switchgrass reference genome), was 2453 cM. Similarly, the map length by Liu et al. (2012) based on simple sequence repeat (SRR) markers was 2085 cM, while that of Lowry et al. (2015) and Milano et al. (2016) based on ddRADseq markers were 2200 and 2289 cM, respectively. The separate male and female maps by Serba et al. (2013) spans 1508 (cultivar Summer) and 1733 (cultivar Alamo) cM, respectively, and by Okada et al. (2010) spans 1515 (cultivar Alamo) and 1935 (cultivar Summer) cM, respectively. Exceptions include Serba et al. (2013), which reported 17 linkage groups in male parent map. Similarly in the maps by Okada et al. (2010) and Liu et al. (2012), the markers that belong to the same chromosome but separated into two clusters were grouped together by manually adjusting the LOD threshold and/or recombination frquencies. Most of the discrepancies in size between different published maps appear due to the marker type, the number of markers, and/or population type. Of all the studies referenced here, the linkage map in this study has the highest marker density, possibly increasing the length of the genetic map.

**Table 5:** Homologs and orthologs of genes, proteins and transcription factors related to freezing and cold tolerance within 1.5 LOD interval of the identified QTL.

QTL	Traits	Best hit transcript	*Panicum virgatum* v4.1	*Arabidopsis thaliana* TAIR10	*Oryza sativa* v7_JGI	*Sorghum bicolor* v3.1.1	*Setaria italica* v2.2	*Zea mays* 5b+	Gene/protein	Reference
1K.3	Tiller number −5°C	Pavir. 1KG002600	Pavir.1KG005600 Pavir.1NG002500	AT3G47450	LOC_Os02g01440	Sobic.004G003900	Seita.1G003800	GRMZM2G384293	NOA1	Zhao et al., 2009
			Pavir.1KG006900 Pavir.1NG003500 Pavir.4NG002900 Pavir.4NG005800	AT5G62390	LOC_Os02g01520 LOC_Os06g01500	Sobic.004G004600 Sobic.010G003300	Seita.1G004500 Seita.4G003300	GRMZM2G041765 GRMZM2G472346	BAG7	Doukhanina et al., 2006; Williams et al., 2010
			Pavir.1KG010400 Pavir.1KG010900	AT1G08090 AT1G08100 AT3G45060 AT5G60770 AT5G60780	LOC_Os02g02170 LOC_Os02g02190	Sobic.004G009200 Sobic.004G009400 Sobic.004G009500	Seita.1G114900 Seita.1G115400 Seita.1G115700 Seita.1G115800 Seita.1G116000 Seita.1G116100	GRMZM2G010251 GRMZM2G010280 GRMZM2G163866	NRT2	Kim et al., 2009; Deshpande et al., 2016
5K.22	LT_50	Pavir. 5KG046800	Pavir.5KG053700 Pavir.5NG036700 Pavir.9NG627200 Pavir.J620200	AT2G19810 AT4G29190	LOC_Os01g09620 LOC_Os05g10670	Sobic.003G034400 Sobic.009G072100	Seita.3G111700	GRMZM2G173124 GRMZM5G801627 GRMZM5G853245	ZF-CCCH	Xin et al., 2007
			Pavir.5KG057400 Pavir.5NG054300		LOC_Os01g09080	Sobic.003G037500	Seita.5G132700	GRMZM2G083350 GRMZM2G176489	WRKY	Ross et al., 2007
			Pavir.5KG060800 Pavir.5NG057600 Pavir.2KG564000 Pavir.2NG607700	AT1G22770	LOC_Os01g08700	Sobic.003G040900	Seita.5G129500	GRMZM2G107101 GRMZM5G844173	GI	Cao et al., 2005; Fornara et al., 2015
			Pavir.5KG070500 Pavir.5KG298900 Pavir.5KG358300 Pavir.5NG069400 Pavir.5NG073300 Pavir.5NG240300	AT3G01540 AT5G14610	LOC_Os01g07740 LOC_Os01g36860 LOC_Os11g46240	Sobic.003G050700 Sobic.003G187400 Sobic.005G220000	Seita.5G121700 Seita.5G197900 Seita.8G239700	GRMZM2G066440 GRMZM2G077125 GRMZM2G078826 GRMZM2G703415	DRH1	Baruah et al., 2017
			Pavir.1KG135200 Pavir.3KG100600 Pavir.5KG078200 Pavir.5NG079300	AT1G75490 AT2G38340 AT2G40340 AT2G40350 AT3G11020 AT5G05410 AT5G18450	LOC_Os01g07120 LOC_Os08g45110	Sobic.003G058200 Sobic.007G162700	Seita.3G065700 Seita.5G115000	GRMZM2G028386 GRMZM2G156737 GRMZM5G838809	DREB2A	Maruyama et al., 2009; Hu et al., 2011
5N.26	Tiller number −9°C	Pavir. 5NG032600	Pavir.5KG051700 Pavir.5NG054100	AT1G64280 AT4G26120	LOC_Os01g09800	Sobic.003G032000	Seita.5G137400	GRMZM2G077197 GRMZM2G077197	NPR1	Yang et al., 2010
			Pavir.5NG022200			Sobic.003G015100	Seita.5G018600	GRMZM2G061005	GH3 gene family	Du et al., 2012
6K.102	Tiller emergence days	Pavir. 6KG368900	Pavir.6KG314100 Pavir.6KG367400	AT2G44730 AT3G24860	LOC_Os08g37810	Sobic.007G225600	Seita.6G188700	GRMZM2G379179 GRMZM5G850092	MYB	Shinozaki et al., 2003
6N.47	Tiller emergence days	Pavir. 6NG191500	Pavir.6NG175100 Pavir.8KG323600 Pavir.9NG040200 Pavir.9NG077100	AT2G15970 AT3G50830	LOC_Os03g55850	Sobic.001G077000	Seita.9G077400	GRMZM2G040030 GRMZM2G052423	COR47	Sharma et al., 2016; Sperotto et al., 2018
			Pavir.6NG183600	AT4G13700	LOC_Os08g17784	Sobic.007G091100	Seita.6G105500	GRMZM2G014193	Purple acid phosphatase	Sperotto et al., 2018
			Pavir.5NG329600 Pavir.6NG205000 Pavir.9NG036500		LOC_Os03g60560	Sobic.001G035100	Seita.9G034200	GRMZM2G002805 GRMZM2G061626 GRMZM2G385575	C2H2 zinc finger	Sperotto et al., 2018
			Pavir.6KG137900 Pavir.6NG181800	AT5G63110	LOC_Os08g25570	Sobic.007G114000	Seita.6G118400	GRMZM2G136067	HDA6	To et al., 2011
			Pavir.1KG237100 Pavir.3KG494700 Pavir.3NG318000 Pavir.5KG619900 Pavir.5NG583900 Pavir.6NG205600 Pavir.9KG460200 Pavir.9KG461600 Pavir.9KG488300 Pavir.9KG489600 Pavir.9KG490000 Pavir.9KG490100 Pavir.9KG490200 Pavir.9KG491000 Pavir.9KG496400 Pavir.9NG440600 Pavir.9NG696800 Pavir.9NG697800 Pavir.J594700 Pavir.J684500	AT1G16030 AT1G56410 AT3G09440 AT3G12580 AT5G02490 AT5G02500	LOC_Os01g62290 LOC_Os03g16860 LOC_Os03g16880 LOC_Os03g16920 LOC_Os03g60620 LOC_Os05g38530 LOC_Os11g08460 LOC_Os11g47760	Sobic.001G418600 Sobic.001G419000 Sobic.001G419200 Sobic.001G419300 Sobic.001G419400 Sobic.001G419700 Sobic.001G419801 Sobic.001G419900 Sobic.001G420100 Sobic.003G350700 Sobic.006G055600 Sobic.008G136000 Sobic.009G163900	Seita.3G216900 Seita.3G327900 Seita.4G060500 Seita.5G376100 Seita.8G225000 Seita.8G225900 Seita.9G033500 Seita.9G451500 Seita.9G451900	AC209784.3_FGP007 GRMZM2G056039 GRMZM2G106429 GRMZM2G145275 GRMZM2G310431 GRMZM2G340251 GRMZM2G366532 GRMZM2G428391 GRMZM5G802801	HSP70	Williams et al., 2010; Sung et al., 2001
9k.80	Tiller number −11°C	Pavir. 9KG370500	Pavir.2KG534500 Pavir.2NG647400 Pavir.9KG412000 Pavir.9NG557600	AT3G06483	LOC_Os03g25400 LOC_Os07g44330 LOC_Os07g44330	Sobic.001G360000 Sobic.002G390500	Seita.2G405900 Seita.9G392000	AC217975.3_FGP001 GRMZM2G030139 GRMZM2G107196	Dehydrogenase kinase	

### QTL for Freezing Tolerance and Comparative Study in Other Species

A genomic comparison of loci within 1.5 LOD of the identified QTL was conducted with annotated genes from *Arabidopsis thaliana, Oryza sativa* (rice), *Setaria italica* (foxtail millet)*, and Sorghum bicolor* (sorghum) and *Zea mays* (maize). A summary of the most promising freezing tolerant genes/transcripts of the identified QTL and their orthologs with *Arabidopsis*, rice, sorghum, foxtail millet, and maize are listed in Table 5. The QTL and their potential freezing tolerant orthologs are discussed, in their order of importance.

The cold responsive gene (COR47), an ortholog of known cold-tolerance genes, resides within the 1.5 LOD interval of QTL 6N.47. The COR47 gene is known to be induced by overexpression of the transcription factor CBF1, and has been reported as an enhancer of freezing tolerance in switchgrass (Pavir.6NG175100) (Sharma et al., 2016), rice (LOC_Os03g55850) (Sperotto et al., 2018) and sorghum (Fiedler et al., 2016) (Table 5). Along with COR, heat shock protein HSP70, a regulator of heat stress (Sung et al., 2001; Williams et al., 2010) is co-located with this QTL. HSP70 might have an important role in the recovery of freezing treated samples, which experience a considerable amount of heat stress during their transition from freezing to regrowth temperature. Other cold-tolerance genes with this QTL encode histone deacetylase (To et al., 2011), purple acid phosphatase (Sperotto et al., 2018), and C2H2 Zinc-finger (Sperotto et al., 2018). The QTL 6N.47 had the highest LOD among all significant QTL and explained 15.8% of phenotypic variation for tiller emergence days (Table 4). Therefore, this QTL could be considered as a major freezing tolerance QTL in switchgrass.

Genes encoding a zinc finger-CCCH type protein (Xin et al., 2007), WRKY (Ross et al., 2007; Tao et al., 2011), GIGANTEA (Cao et al., 2005; Fornara et al., 2015) and DEAD-box ATPase-RNA-helicase (DRH1) (Mahajan and Tuteja, 2005; Baruah et al., 2017) are co-located with QTL 5K.22, a QTL determined by LT_50_. An ortholog of the dehydration-responsive element-binding protein 2A (DREB2A) (Table 5) in *Arabidopsis* (Maruyama et al., 2009; Hu et al., 2011), rice (Dubouzet et al., 2003), and foxtail millet (Lata and Prasad, 2014) is also located in this region. Expression of DREB2A is induced by dehydration and several studies mentioned its cognate *cis*-element, DREB1, which is involved in the regulation of low-temperature stress. However, a recent molecular study in *Arabidopsis* has suggested DREB2A to also be involved in the response to cold (Nakashima et al., 2014). At least one homolog of all five genes mentioned above is located on chromosome 1 of rice and chromosome 8 of maize (Table 5), indicating that a synthetic region for freezing tolerance is conserved across closely related species.

Dehydrogenase kinase enzyme (Goodstein et al., 2011) is co-located with QTL 9K.80. Milano et al. (2016) identified one QTL related to tiller number among others closely located to this QTL. Serba et al. (2013) and Lowry et al. (2015) identified several major switchgrass biomass QTL in this chromosome, although, whether these QTL fall under the same QTL confidence interval of this study remains to be determined. Since, biomass yield had a strong correlation with tiller number (Bhandari et al., 2011), the impact of this QTL on these important agronomic traits could be associated with its ability to tolerate freezing temperatures.

Orthologs of potential freezing tolerance genes co-localized within QTL 1K.3 include: a P-loop containing nucleoside triphosphate hydrolases superfamily protein (NOA1) (Zhao et al., 2009; Costa-Broseta et al., 2018), nitrate transporter (NRT2) (Kim et al., 2009), and BCL-2-associated athanogene 7 (BAG7) (Doukhanina et al., 2006) (Table 5). NOA1 and NRT2 are down-regulated in response to cold stress in *Arabidopsis*, while BAG7 is upregulated and is thought to be involved as cryo-protectants (Williams et al., 2010). Moreover, at least one ortholog of these three genes is within a 5 MB region of chromosome 2 in rice and chromosome 4 in sorghum, where major early cold tolerance QTL were identified previously (Andaya and Mackill, 2003; Lou et al., 2007; Knoll et al., 2008; Burow et al., 2011).

The genes co-located with QTL 5N.26 include: a rice GH3 gene family member (OsGH3-2) (Du et al., 2012) and an ortholog of the *Arabidopsis* gene NPR1 (Yang et al., 2010) (Table 5). In response to cold, the expression of OsGH3-2 is suppressed due to modulation of endogenous free indole-3-acetic acid (IAA) and abscisic acid (ABA). NPR1 signals for response to external stimuli and is involved in both the abiotic and biotic stress response pathway. The *Arabidopsis* transcription factor MYB (AT2G44730), orthologous to Pavir.6KG314100 and Pavir.6KG367400, is co-located with QTL 6K.102. MYB binds to promoter regions in response to dehydration and helps in the accumulation of endogenous ABA, suggesting its role in cold tolerance (Shinozaki et al., 2003).

Although we mentioned several potential orthologs of the cold-tolerance gene mostly from *Arabidopsis* and rice, it must be noted that these species are annual while switchgrass is perennial. Many of these genes were studied through transcriptome analysis, either during the seedling or early developmental stage. This is different from our study because we were interested in finding QTL associated with freezing tolerance during the dormant stage. It is unfortunate that cold tolerance and freezing tolerance are often incorrectly used synonymously, even though they denote different concepts (Hincha and Zuther, 2014). Freezing damage is a physical process caused by osmotic dehydration, triggered by extracellular ice formation while low-temperature or cold damage is a subjective or relative term, a direct effect of temperature (Hincha and Zuther, 2014). It is very important to understand this concept because most of the switchgrass cultivars are usually not affected at non-freezing low temperatures, which may not be true in rice and *Arabidopsis*. However, the comparison across species still could provide insights into the historically conserved genetic mechanism associated with freezing and low-temperature stress (Sandve et al., 2011). Further assessment of these QTL, either through fine mapping or functional analysis with mutants, would be necessary before using them in a marker-assisted breeding program.

## Conclusion

Reported studies for improving winter survival in switchgrass are based on phenotypic selection methods and this is the first report of QTL for freezing tolerance within this species. Low-temperature or freezing stress is one of the major abiotic environmental stresses affecting survival of the lowland ecotype of switchgrass at northern latitudes of the United States (Lemus et al., 2002; Milano et al., 2016). Accurate phenotyping for cold tolerance in the field limits the effectiveness of selection because plant survival is highly dependent on weather conditions which generate freezing stresses. As such, field-based selection requires something of an optimal environment, not too cold as to result in nearly complete mortality, but sufficiently cold to kill non-hardy genotypes. The results of this study will be helpful for following two aspects: (1) the estimated LT_50_ temperature will be helpful in determining the threshold temperature for conducting future research related to winter survival and (2) the detected QTL will serve as valuable genetic resources for understanding the genetic basis of freezing tolerance and improving lowland switchgrass toward the development of superior cultivars.

## Author Contributions

HP and MC designed the experiments. HP collected the phenotypic data and performed statistical and QTL analysis. MS and CB performed the genotyping using exome-capture sequencing technique. HP led the writing of manuscript and MS, CB, SK, and MC participated in writing the manuscript.

## Conflict of Interest Statement

The authors declare that the research was conducted in the absence of any commercial or financial relationships that could be construed as a potential conflict of interest.

## Footnotes

1 https://www.ncdc.noaa.gov

2 https://biotron.wisc.edu/

3 http://www.bioinformatics.babraham.ac.uk/projects/fastqc/

4 https://github.com/broadinstitute/picard/releases/tag/2.7.2

## Supplementary Material

TABLE S1 The genotype data of selected 1618 markers and 341 F2 progenies used in QTL analysis. The genotype are coded as cross pollinated (CP) format based on JoinMap 4.1 (Van Ooijen, 2006). < hkxhk > : F2 type, segregating 1:2:1, hh and kk mean homozygous to each of the grandparents Summer or Ellsworth and hk means heterozygous. < lmxll > and < nnxnp > : both BC1-type, segregating 1:1.

TABLE S2 The genetic map of 1618 markers in 18 linkage groups. The physical distance is in base pairs (bp) and map distance are in Kosambi map units (cM). The markers are grouped by chromosomes.

TABLE S3 Best linear unbiased predictor (BLUP) of phenotypic data for all traits used in QTL analysis. LT-50 are in °C while all other traits are measured as the number (n). NA, Not available because the genotype was dead in field, [.] = The BLUP was not estimable.

FIGURE S1 The minimum air temperature during the acclimation period recorded in the nearby station (Madison Dane County Regional Airport, WI, United States, https://www.ncdc.noaa.gov). The horizontal dashed line represents the freezing point below which the rhizomes in cold frames were covered with plastic tarps.
