# Supplementary figures and images for: Quantitative Trait Loci for Freezing Tolerance in a Lowland x Upland Switchgrass Population
Source: Front Plant Sci. 2019 Mar 29;10:372. doi: 10.3389/fpls.2019.00372 (PMC6450214; doi:10.3389/fpls.2019.00372)

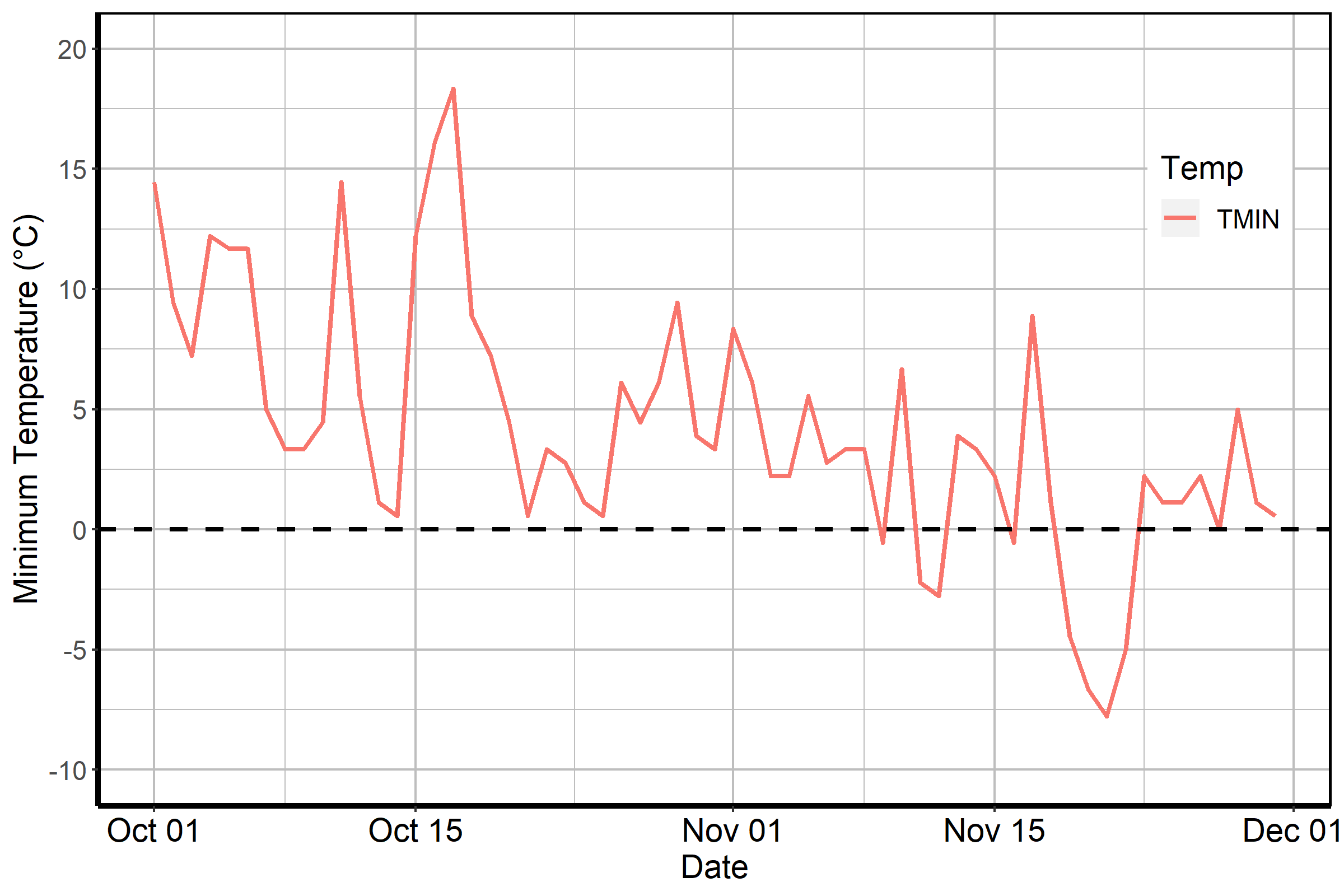

Supplement: FIGURE S1 — The minimum air temperature during the acclimation period recorded in the nearby station (Madison Dane County Regional Airport, WI, United States, https://www.ncdc.noaa.gov). The horizontal dashed line represents the freezing point below which the rhizomes in cold frames were covered with plastic tarps. [file Image_1.TIFF]
